# Physical functioning associated with life-space mobility in later life among men and women

**DOI:** 10.1186/s12877-022-03065-9

**Published:** 2022-04-26

**Authors:** Sofi Fristedt, Ann-Sofi Kammerlind, Eleonor I. Fransson, Marie Ernsth Bravell

**Affiliations:** 1 School of Health and Welfare, Jönköping University, Box 1026, 551 11 Jönköping, Sweden; 2 Department of Health Sciences, Faculty of Medicine, Lund University, Box 157, 221 00 Lund, Sweden; 3 Futurum, SE-551 85 Linköping, Region Jönköping County Sweden; 4 Department of Health, Medicine and Caring Sciences, Linköping University, SE-581 83 Linköping, Sweden

**Keywords:** Community mobility, Dizziness, Hearing, Lung function, Vision, Older people

## Abstract

**Background:**

Life-space mobility is defined as the ability to access different areas extending from the room where the person sleeps to places outside one’s hometown. Life-space mobility is vital to support performance of daily life activities and autonomous participation in social life. However, there is a dearth of research that investigates a wider range of physical functions and functioning in relation to life-space mobility rather than just single aspects. Thus, the purpose of the present study was to identify and describe several measures of physical functioning associated with life-space mobility among older men and women.

**Methods:**

Data used in this study was derived from the OCTO 2 study, a population-based study of health, functioning and mobility among older persons (*n* = 312) in Sweden. Associations between Life-Space Assessment (LSA) total score and age, sex, Short Physical Performance Battery (SPPB), dizziness, lung function i.e. Peak Expiratory Flow (PEF), grip strength, self-rated vision and hearing were analysed through bivariate and multivariate regression models.

**Results:**

The bivariate models showed that life-space mobility was significantly associated with sex, but also age, SPPB, PEF and grip strength in the total group as well as among men and women. In addition, hearing was significantly associated with life-space mobility among women. Those factors that were statistically significant in the bivariate models were further analysed in multivariable models for the total group, and for men and women separately. In these models, sex, grip strength and SPPB remained significantly associated with life-space mobility in the total group, as well as SPPB among both men and women.

**Conclusion:**

Sex, physical function in terms of physical performance measured by SPPB (balance, gait speed and chair stand), and grip strength are associated with life-space mobility. Consequently, these factors need to be considered in assessments and interventions aiming to maintain mobility in old age.

## Introduction

Life-space mobility can be defined as the ability to access different areas defined as life-spaces extending from the room where the person sleeps, to places outside one’s hometown [1, 2]. Life-space mobility is vital to support performance of daily life activities and autonomous participation in social life [3–5]. The importance of life-space mobility remains, even though many virtual mobility options today are available to satisfy a person’s social and practical needs in daily life. In fact, being able to physically move oneself to places for activities dependent on face-to-face interaction has some clear advantages [6]. For example, the possibility to actively visit other people or arenas to access social activities, rather than being dependent on others visiting you, is likely to promote a sense of belonging and social inclusion, and may decrease loneliness [7]. Not least, being able to transfer or transport oneself supports the performance of activities important to satisfy basic human needs. These could be activities related to acquiring food and shelter or to remaining engaged in society and social life [8, 9]. In addition, life-space mobility often includes physical activity, with its well-documented positive implications on human health [10].

In later life, however, there is a risk of limitations in life-space mobility for several reasons such as driving cessation [11], lack of transportation, or environmental demands [12, 13]. This risk is evident also in Sweden and other Western countries despite general good accessibility in public environments and in housing [14]. Furthermore, previous studies have identified risks of developing life-space mobility restrictions related to physical performance [15], hearing difficulties [16] or disability in Activities of Daily Living (ADL) [17], and life-space mobility has been related to gait quality [18]. Recent studies also show that cognitive limitations may be detected by gait speed and life-space mobility assessments [19] and that physical activity levels during hospitalisation are associated with life-space mobility in stroke survivors following discharge [20]. These studies address single impairments, (e.g., physical performance or hearing), but it is rare that a wider range of physical functions and functioning associated with life-space mobility (e.g., physical performance and hearing etc.) among older men and women is considered in the same study. More knowledge about older people’s mobility, and the association of different factors related to mobility limitations is needed to guide and evaluate interventions aiming to promote life-space mobility in later life. Thus, the purpose of the present study was to identify and describe physical functioning associated with life-space mobility among older men and women. The hypothesis was that a range of physical functioning aspects are associated with life-space mobility among Swedish older men and women.

## Method

### Participants and data collection

Data used in this study was derived from the OCTO 2-study, a population-based study that was set up with the main aim of investigating health, social networks, physical activity, physical and mental functioning and mobility among older persons in Sweden [21–23]. The study was approved by the regional ethical committee in Linköping, Sweden (#225–08), and the research was conducted in accordance with Swedish law and the Declaration of Helsinki. The randomly selected sample was obtained from a population register, including men and women, 75, 80, 85, or 90 years-old, living in Jönköping County Council, Sweden, in 2009 and 2010. Persons for whom we had information from relatives or staff that they suffered from dementia, were excluded from the study. The original aim was to include 450 participants, based on a random sample of 600 individuals. However, an additional random sample was conducted to reach the final sample of 327 participants who voluntarily chose to participate and gave their informed written consent. Trained, registered research nurses performed data collection, i.e. interviews and tests of physical performance, during home visits. During the interviews, the participants responded to a large set of questions on subjects including lifestyle, social relationships, ADL, health, physical functioning and mobility. They also reported on personal characteristics such as their marital status, type of housing, type of living area, use of assistive devices and whether they currently drove a car or not. The present study excluded participants living in nursing homes (*n* = 10), and those who had not responded to the Life-Space Assessment (LSA, see below *n* = 5*)* yielding an analytical sample of 312 community-dwelling participants. Most of the participants received no care (61%), 19% received informal care only, and 20% received formal care.

### Instruments and variables

Life-Space Assessment (LSA) was the outcome of interest in this study. The LSA includes six levels of life-space, ranging from the person’s bedroom (Life-space 0) to places beyond the person’s hometown (Life-space 5) [24]. For each of these six levels, the person is asked how often they have been to that specific life-space area during the last four weeks, and whether they did so independently or needed assistance from another person or equipment. A total LSA score is obtained by multiplying the life-space level reached (1–5) by the value for the frequency of transportation (1–4) for each life-space level, as well as by the value for independence (2, 1.5, or 1), and then summarising the scores of the five levels. The total score can range from 0 (totally confined to bedroom) to 120 (independent, with daily out-of-town mobility).

To identify possible associations with life-space mobility, age in years (75, 80, 85 and 90 years of age) and sex (men and women) were used as independent variables together with the OCTO 2 variables that targeted physical functioning, namely:

- Short Physical Performance Battery (SPPB), utilised to assess older persons physical performance [25], was used as a "total score” summarising the three different subtest scores, i.e. “total balance” including a hierarchical test of standing balance, “gait speed” during a three-metre walk and “chair stand” including five repetitive chair stands, ranging from 1 (poor ability) to 12 (good ability).
- Self-reported dizziness (1 = none/2 = mild/3 = substantial) was based on two questions. All participants were asked if they experienced dizziness (yes/no). Persons with perceived dizziness also responded to the University of California Los Angeles Dizziness Questionnaire (UCLA-DQ) [26] including questions about the frequency and intensity of dizziness, rated from 1 (least severe) to 5 (most severe) respectively. Subjects who reported the least severe response choice for the items of frequency (rarely) and/or intensity (very mild) were categorised as having mild dizziness, and subjects reporting more severe symptoms were categorised as having substantial dizziness [27]. For the linear models the variable was dichotomised none/mild vs. substantial.
- Peek Expiratory Flow (PEF) measured in litres per minute. The participants blow twice forcefully into a Peak Flow Metre (best result out of the two recorded, z-standardised for men and women).
- Grip strength measured using Vigorimeter® [28] measured in kilopascal (kpa) (best result out of possibly three, for dominant hand, z-standardised for men and women). The large ball was used for men and a medium-sized ball was used for women.
- Self-rated vision with or without glasses (1 = very good vision/2 = good vision/3 = fairly good/4 = bad/5 = very bad). For the linear models the variable was dichotomised good (1–2) vs. bad (3–5).
- Self-rated hearing with or without hearing aid (1 = very good hearing/2 = good hearing/3 = fairly good/4 = bad/5 = very bad). For the linear models the variable was dichotomised good (1–2) vs. bad (3–5).

### Analysis

SPSS version 26 was used for the statistical analysis. Non-parametric statistics (Mann–Whitney U-test and χ^2^) were used to identify differences between men and women in background characteristics, life-space mobility and physical functioning. The independent variables, as listed above, were first entered into bivariate linear regression models using the LSA total score as the dependent variable, to identify factors significantly associated with life-space mobility. This was done for the total group, as well as for men and women separately. In the next step, factors identified as significant in the bivariate models, were entered into multivariable linear regression models for the total group, as well as for men and women. Unstandardised regression coefficients and 95% confidence intervals were derived by the regression analyses. A *p*-value < 0.05 was considered as statistically significant.

## Results

Considering the characteristics of the sample (Table 1), some differences were statistically significant when comparing men and women. For example, men were more often married, and women more often widowed. Compared to women, men were also more often living in their own house and still driving a car.

**Table 1:** Characteristics of the study sample and group comparison (*n* = 312)

Variable	Total group	Men	Women	*p*-value
Sex, n (%)				
Males	147 (47)	-	-	
Females	165 (53)	-	-	
Age, n (%)				0.021^1^
75	107 (34)	51 (35)	56 (34)	
80	94 (30)	55 (37)	39 (23)	
85	67 (22)	26 (18)	41 (25)	
90	44 (14)	15 (10)	29 (18)	
Marital status, n (%)				< 0.001^1^
Married	164 (53)	114 (78)	50 (30)	
Widows/widowers	119 (38)	22 (15)	97 (59)	
Never married	13 (4)	4 (3)	9 (6)	
Divorced	16 (5)	7 (5)	9 (6)	
Type of housing, n (%)				< 0.001^1^
Own house	157 (50)	94 (64)	63 (38)	
Apartment	155 (50)	53 (36)	102 (62)	
Type of living area, n (%)				0.045^2^
Living in town > 5000 inhabitants	205 (66)	87 (60)	118 (72)	
Living in village 200–5000 inhabitants	68 (22)	41 (28)	27 (16)	
Living in small village < 200 inhabitants	13 (4)	7 (5)	7 (4)	
Living in the countryside, not in village	25 (8)	12 (8)	13 (8)	
Use of assistive devices, n (%)				0.004^1^
Yes	155 (50)	60 (41)	95 (58)	
No	156 (50)	86 (59)	70 (42)	
Driving a car				< 0.001^1^
Yes	160 (51)	108 (73)	51 (32)	
No	152 (59)	39 (27)	113 (68)	
LSA total score (0–120), mean (SD)	64 (23)	72 (21)	58 (23)	< 0.001^2^
Independent life-space^a^ (0–5), median (Q1-Q3)	4 (2–5)	5 (4–5)	4 (1–5)	< 0.001^2^
Assistive life-space^b^ (0–5), median (Q1-Q3)	5 (4–5)	5 (4–5)	4 (3–5)	< 0.001^2^
Maximal life-space^c^ (0–5), median (Q1-Q3)	5 (5–5)	5 (5–5)	5 (4–5)	0.06^2^
SPPB total score (0–12), median (Q1-Q3)	10 (7–11)	10 (8–12)	9 (7–11)	0.001^2^
total balance (0–4), median (Q1-Q3)	4 (3–4)	4 (4–4)	4 (3–4)	< 0.001^2^
gait speed (0–4), median (Q1-Q3)	3 (2–4)	3 (3–4)	3 (2–4)	< 0.001^2^
chair stand (0–4), median (Q1-Q3)	1 (1–1)	1 (1–1)	1 (1–1)	0.24^2^
PEF mean (SD)	400 (119)	469 (116)	340 (85)	< 0.001^2^
Grip strength mean (SD)	0.55 (0.18)	0.6 (0.15)	0.51 (0.19)	< 0.001^2^
Vision (1–5) median (Q1-Q3)	2 (2–3)	2 (2–3)	2 (2–3)	4.76^2^
good/bad n (%)	275 (88)/36 (12)	127 (86)/20 (14)	148 (90)/16 (10)	
Hearing (1–5) median (Q1-Q3)	3 (3–4)	3 (3–4)	4 (3–4)	0.004^2^
good/bad, n (%)	271 (87)/40 (13)	119 (81)/28 (19)	152 (92)/12 (8)	
Dizziness (1–3) median (Q1-Q3)	1 (1–3)	1 (1–2)	1 (1–3)	0.29^2^
None or mild/substantial, n (%)	217 (70)/95 (30)	98 (67)/49 (33)	119 (72)/46 (28)	

^1^ χ^2 2^ Mann–Whitney U-test ^a^ highest life-space level obtained without any assistance ^b^ highest life-space level reached with help from equipment but not another person ^c^ maximal life-space level indicates the greatest distance travelled irrespective of assistance from equipment and/or another person

The bivariate models showed that life-space mobility was significantly associated with sex, but also age, SPPB, PEF and grip strength in the total group as well as among men and women (Table 2). In addition, hearing was significantly associated with life-space mobility in women.

**Table 2:** Factors associated with life-space mobility in the bivariate linear regression models

	Total group			Men			Women		
	R^2^	B	95% CI for B	R^2^	B	95% CI for B	R^2^	B	95% CI for B
Age in years									
75^1^	0.158			0.093			0.196		
80		-5.52	-11.43;0.40		-4.87	-12.75;3.01		-9.14	**-17.61;-0.67**
85		-13.19	**-19.69;-6.69**		-12.19	**-21.95;-2.43**		-12.44	**-20.72;-4.16**
90		-27.46	**-34.93;-19.99**		-20.80	**-32.70;-8.90**		-28.93	**-38.15;-19.72**
Sex (men = 0; women = 1)	0.090	-13.81	**-18.72;-8.90**	-	-	-	-	-	-
SPPB	0.380	5.02	**4.30;5.75**	0.356	5.15	**4.00;6.29**	0.355	4.46	**3.52;5.41**
PEF (z-stand)	0.057	5.49	**2.99;7.98**	0.058	5.12	**1.73;8.51**	0.067	5.81	**2.44;9.17**
Grip strength (z-stand)	0.099	7.23	**4.78;9.68**	0.094	6.54	**3.21;9.87**	0.120	7.80	**4.51;11.09**
Vision (good = 0; bad = 1)	0.001	1.62	-6.37; 9.62	0.003	3.17	-6.98;13.32	0.002	-2.97	-14.65;8.71
Hearing (good = 0; bad = 1)	0.001	1.98	-5.66;9.63	0.003	2.90	-5.97;11.76	0.016	-10.72	-23.93;2.49
Dizziness (none/mild = 0; substantial = 1)	0.004	3.49	-2.51;9.48	0.000	-0.20	-8.34;7.94	0.022	7.53	-0.20;15.23

Statistically significant associations on a 5% significance level are marked as bold. ^1^ Age group 75 years used as reference

When further analysing the factors that were statistically significant according to the bivariate models (Table 2) for total group sex, grip strength and SPPB remained significant in the multivariable model for the total group. However, in the gender-specific models only SPPB remained significant (Table 3).

**Table 3:** Factors associated with life-space mobility in the multivariable linear regression models

	Total group			Men			Women		
	Adj R^2^ = 0.414	B	95% CI for B	Adj R^2^ = 0.358	B	95% CI for B	Adj R^2^ = 0.359	B	95% CI for B
Age in years									
75^1^	0.125			0.095			0.154		
80		-0.47	-5.37;4.43		3.30	-3.48;10.09		-4.68	-11.98;2.63
85		-3.71	-9.23;1.82		-4.25	-12.60;4.09		-4.02	-11.60;3.56
90		-6.00	-13.09;1.09		-2.17	-12.92;8.59		-8.54	-18.19;1.10
Sex (men = 0; women = 1)		-8.05	**-12,03;-4.07**		-	-		-	-
SPPB		4.09	**3.25; 4.93**		4.80	**3.46; 6.13**		3.71	**2.61; 4.81**
PEF (z-stand)		-0.25	-2.39; 1.88		-0.12	-3.08; 2.84		-0.54	-3.70; 2.63
Grip strength (z-stand)		2.60	**0.49; 4.71**		2.30	-0.60; 5.31		3.12	-0.07; 6.18

significant associations marked as bold ^1^ Age group 75 years used as reference

## Discussion

The present study identified and described physical functioning associated with life-space mobility among older men and women. A range of physical functioning aspects, namely physical performance, lung function, grip strength, vision, hearing and dizziness were considered. Together with sex, only the combined measure of physical performance (SPPB, including balance, gait speed and chair stand) and grip strength were connected to life-space mobility. In the gender-specific multivariable models only SPPB was connected to life-space mobility. The women in the group had a significantly lower LSA total score compared to the men, although the total group had a high total score. The significantly lower LSA total score for women may be due to the fact that women in this study drove their own car to a lesser extent than the men. This may be disadvantageous to women since the car brings unique and more flexible opportunities to reach more distant life-spaces in later life [29]. Similar findings have also been noted before in similar populations [8]. However, there were also other significant differences between men and women in our sample that may have impacted on our findings, for example the women used assistive devices to a greater extent than the men in the study.

It is not surprising that physical performance (SPPB) that supports walking ability was connected to LSA. Walking represents a common link of the travel chain (walking from the home to the car or bus stop, from the car or bus to the final destination etc.) and is thereby vital to reach different life-spaces. This result is also similar to the result of a previous study showing that poorer physical performance is associated with more life-space mobility restrictions, as well as has an indirect effect on sense of autonomy outdoors [15].

While restricted life-space mobility could be an early sign of vulnerability [17], our findings identified that another proxy of frailty, grip strength, also in turn was associated with life-space mobility in the total sample. However, gender differences observed in previous studies [30–32] did not apply to our study. For example, Sternäng et al. [30], found gender differences, not only in grip strength, but in the type of factors associated with grip strength performance and decline. More specifically, grip strength decline was associated with stress, smoking and dementia for women, and marital status, mean arterial pressure, physical activity at work and having a chronic disorder for men. de Araújo Amaral et al. [31] concluded that factors associated with low hand grip strength differs between sexes, and was associated with physical activity for example only among women. And finally, Ernsth Bravell et al. [32] women demonstrated more difficulties with fine motor functions, and fine motor functions were predictive of mortality only for women, not for men.

Fine motor function as in grip strength may be seen as a proxy for frailty or vulnerability, that in the long run may cause a cascade of severe outcomes, such as falls, fractures, and hospitalisations, that lead to limited life-space mobility and mortality. Also, both female gender and lower physical function are associated with fear of falling and fear of falling-associated activity restriction [33], which may also influence life-space mobility [34]

This reasoning also implies that grip strength, as well as lung function (PEF) would play an even more significant role in a less healthy sample of older adults. Several studies i.e. [30] conclude that women demonstrate lower grip strength in comparison to men beyond the expected differences. As mentioned above, beyond the general sex differences in muscle strength across ages, the lower grip strength is usually explained by the fact that women have more chronic conditions that will impact functioning in different ways. Men, on the other hand, are less likely to have chronic conditions that impact motor functioning, but suffer more from acute, fatal, diseases [35, 36]. Factors related to motor function, such as grip strength, are important to notice, given that they not only affect daily living, but also the life-space mobility and mortality. In contrast to a previous study [16], hearing was only associated with life-space mobility in women in the bivariate models. Unlike that previous study, our data did not consider the magnitude, but rather the patient reported presence of hearing problems.

It can be of value to identify different types of functioning before they result in reduced mobility on an individual level. The results also highlight the importance of considering the gender differences both in practical settings and when designing future studies in the area. Even though chronic conditions, mostly present among women, are not considered life-threatening, they may severely affect mobility and in the long run, mortality.

### Limitations

The present study uses already collected data to respond to the purpose of the present study. Although the existing database included some relevant variables, it would be interesting as well as relevant to include other variables representing physical functioning in future studies, to further explore how a wider range of physical functioning is related to life-space mobility. It should be noted that the men and women included differed significantly in several background characteristics (marital status, living area, car driving) and it would be relevant to further study these issues based on a more homogeneous sample of men and women. It should also be noted that the sample size in the stratified analyses by sex was rather limited in the present study, leading to low power in these analyses. Similar to many other gerontological studies, the database includes a rather healthy sample, which could explain some of our findings as described above and may also limit the generalisability of our findings.

## Conclusion

Physical function in terms of physical performance (balance, gait speed and chair stand) and grip strength are associated with life-space mobility. Consequently, these factors need to be considered in assessments and interventions aiming to maintain mobility in old age.

## Data Availability

Data used in the present study are available upon request from the corresponding author at sofi.fristedt@ju.se.

## Abbreviations

LSA: Life-Space Assessment
SPPB: Short Physical Performance Battery
